# Improving Soybean Germination and Nodule Development with Nitric Oxide-Releasing Polymeric Nanoparticles

**DOI:** 10.3390/plants14010017

**Published:** 2024-12-25

**Authors:** Ana Cristina Preisler, Giovanna Camargo do Carmo, Rafael Caetano da Silva, Ana Luisa de Oliveira Simões, Juliana de Carvalho Izidoro, Joana Claudio Pieretti, Roberta Albino dos Reis, André Luiz Floriano Jacob, Amedea Barozzi Seabra, Halley Caixeta Oliveira

**Affiliations:** 1 Department of Animal and Plant Biology, Londrina State University, Londrina 86057-970, PR, Brazil; preislerac@gmail.com (A.C.P.); giovannacdcarmo@gmail.com (G.C.d.C.); ana.luisa.oliveira@uel.br (A.L.d.O.S.); 2 Department of Agronomy, Londrina State University, Londrina 86057-970, PR, Brazil; 3 Department of Biodiversity Conservation, Institute of Environmental Research, São Paulo 04301-902, SP, Brazil; rafael.caetano94@gmail.com; 4 Center for Natural and Human Sciences (CCNH), Federal University of ABC (UFABC), Santo André 09210-580, SP, Brazil; julianaizidoro@yahoo.com.br (J.d.C.I.); joana.pieretti@ufabc.edu.br (J.C.P.); roberta.reis@ufabc.edu.br (R.A.d.R.); andre.jacob@aluno.ufabc.edu.br (A.L.F.J.); amedea.seabra@ufabc.edu.br (A.B.S.)

**Keywords:** nanocarriers, nodule, S-nitrosoglutathione, nitric oxide, chitosan nanoparticles, alginate nanoparticles

## Abstract

Nitric oxide (NO) is a multifunctional signaling molecule in plants, playing key roles in germination, microbial symbiosis, and nodule formation. However, its instability requires innovative approaches, such as using nanoencapsulated NO donors, to prolong its effects. This study evaluated the impact of treating soybean (*Glycine max*) seeds with the NO donor S-nitrosoglutathione (GSNO), encapsulated in polymeric nanoparticles, on the germination, nodulation, and plant growth. Seeds were treated with free GSNO, chitosan nanoparticles with/without NO (NP CS-GSNO/NP CS-GSH, where GSH is glutathione, the NO donor precursor), and alginate nanoparticles with/without NO (NP Al-GSNO/NP Al-GSH). Chitosan nanoparticles (positive zeta potential) were smaller and released NO faster compared with alginate nanoparticles (negative zeta potential). The seed treatment with NP CS-GSNO (1 mM, related to GSNO concentration) significantly improved germination percentage, root length, number of secondary roots, and dry root mass of soybean compared with the control. Conversely, NP CS-GSH resulted in decreased root and shoot length. NP Al-GSNO enhanced shoot dry mass and increased the number of secondary roots by approximately threefold at the highest concentrations. NP CS-GSNO, NP Al-GSNO, and NP Al-GSH increased S-nitrosothiol levels in the roots by approximately fourfold compared with the control. However, NP CS-GSNO was the only treatment that increased the nodule dry mass of soybean plants. Therefore, our results indicate the potential of chitosan nanoparticles to improve the application of NO donors in soybean seeds.

## 1. Introduction

The establishment of a crop in the field is contingent upon the successful completion of the germination and emergence phases, as these steps serve as the foundation for subsequent growth and development. It is therefore of great consequence that only high-quality seeds are used, as these are capable of demonstrating maximum germination potential and vigor [1]. In this context, rapid and synchronous germination is of great significance, as it promotes greater uniformity in the initial development of plants, enhancing the crop’s competitiveness and resilience against biotic and abiotic stresses, and improving yields in agricultural areas [2].

In the cultivation of soybean [*Glycine max* (L.) Merr.], seed treatment represents a fundamental step in the extension of seed shelf-life and improvement in seedling establishment in the field [3,4,5,6].Inoculation is one of the most widely known and used practices in soybean cultivation, largely due to the crop’s high nitrogen (N) demand. This demand can be met sustainably through the process of biological nitrogen fixation (BNF), which occurs via symbiosis with *Bradyrhizobium*. These bacteria are capable of fixing atmospheric N_2_, converting it into a form that can be assimilated by the plant [7,8]. This plant–microorganism interaction involves the formation of specialized structures, called nodules, where BNF occurs [7,9].

Nitric oxide (NO) is a gaseous molecule with a wide range of functions in plant growth and development, and in plant–microorganism interactions [10]. The role of NO in the germination process has been elucidated, with evidence indicating that it reduces abscisic acid (ABA) levels and stimulates the mobilization of reserves, while also modulating root development [11,12,13,14]. Furthermore, NO plays a role in the formation of specialized structures, such as nodules, and is involved in multiple stages of their development, including recognition, infection, organogenesis, and senescence [15].

However, due to the unstable nature of NO, its exogenous application requires the use of donor molecules, e.g., S-nitrosoglutathione (GSNO), which, although being significantly more stable than NO in its gaseous form, still exhibit relative instability in releasing the NO contingent in environmental conditions. In this context, the nanoencapsulation of NO donors has emerged as a strategy to regulate NO release, protecting the NO donor against premature degradation, thereby enhancing the NO delivery and efficiency to plants [16].

Particles within the nanometric size range are classified as nanoparticles (NPs), which have been utilized in a plethora of applications due to their distinctive physical, chemical, and biological characteristics [16,17]. Their nanoscale dimensions afford a high surface-to-volume ratio, thereby facilitating enhanced reactivity, targeted delivery, and controlled release of substances [16,17]. With regard to polymeric NPs, chitosan and alginate have been identified as particularly promising materials, given their biocompatibility, biodegradability, and functional versatility [18,19,20,21].

Chitosan nanoparticles have been the only polymeric nanoparticles employed for the delivery of NO donors in agricultural applications [16]. The application of chitosan nanoparticles to soybean seeds has been demonstrated to reduce the respiration rate, ethylene content, and lipid peroxidation that occurs during storage. Furthermore, the treated seeds exhibited an enhanced vigor index and germination rate [6]. Steven et al. (2024) [22] also observed that treating soybean seeds with chitosan nanoparticles containing GSNO promoted better germination and seedling growth, due to the positive regulation of gibberellins (GAs) and the negative regulation of ABA.

The current knowledge regarding the interaction between plants and polymeric nanoparticles remains limited. Nanomaterials have been observed to exert a range of effects on plants, including the modification of plant development and induction of oxidative stress. These effects, along with the absorption and transport of nanomaterials by plants, are closely related to their physicochemical properties, including size, composition, and surface charge [21,22,23,24]. For example, Preisler et al. (2022) [25] observed that polymeric nanoparticles with a more positive surface charge exhibited a greater tendency to accumulate on the surface of *Brassica juncea* seeds. Conversely, those with a more negative charge demonstrated a higher rate of absorption, resulting in greater accumulation within the seed over time.

In this context, it is of great importance to assess the efficacy of other biopolymers, such as alginate, to compose nanocarriers of NO donors. As these nanoparticles are novel, further investigation is required in order to determine the characteristics of each particle and to evaluate their performance under conditions that are conducive to their optimal functioning. This would facilitate the development of more effective applications.

The present study aimed to assess the impact of treating soybean seeds with the NO donor GSNO, encapsulated within chitosan or alginate nanoparticles, on the germination process. Furthermore, our secondary objective was to ascertain whether seed treatment with nanoparticles would enhance the nodulation and growth of soybean plants.

## 2. Results

### 2.1. Characterization of Nanoparticles and Kinetics of NO Release

GSH-loaded chitosan nanoparticles (NP CS-GSH) exhibited a mean hydrodynamic diameter of 112.3 nm, a polydispersity index (PDI) of 0.257, and a zeta potential of 20.2 mV. The mean hydrodynamic diameter of GSH-loaded alginate nanoparticles (NP Al-GSHs) was observed to be 228.4 nm, with a PDI of 0.3 and a zeta potential of −15.3 mV. These characteristics indicate a small size, monodisperse size distribution, and good colloidal stability, respectively, of both polymeric nanoparticles, although they show opposite surface charges.

**Figure 1:**
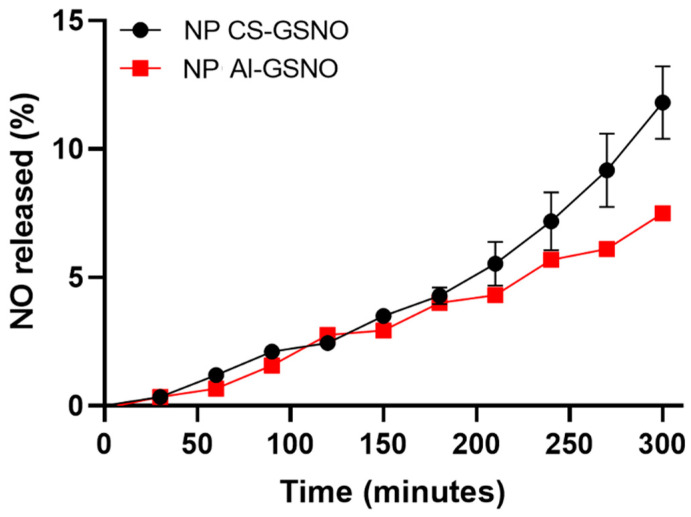
Kinetic curves of NO release from chitosan and alginate nanoparticles loaded with S-nitrosoglutathione (NP CS-GSNO and NP Al-GSNO). Data are expressed as mean ± standard error (n = 3).

Figure 1 illustrates the NO release profile of GSNO-loaded NPs CS and NPs Al over time. In the initial phase, the release rate of both types of nanoparticles was low and comparable, with a duration of approximately 50 min. As time elapsed, a gradual increase in the percentage of NO release was observed for both nanoparticles. Nevertheless, NPs CS exhibited a more pronounced release profile, reaching approximately 14% after 300 min, while NPs Al demonstrated a lower release rate, achieving about 8% at the same time point.

### 2.2. In Vitro Experiment with Soybean

#### 2.2.1. Germination Parameters

Figure S1 shows representative images of soybean seedlings from the different seed treatments. While the results of the ANOVA (Table S1) for NP Al-GSNO did not show statistical significance for the germination percentage, the seed treatment with the other formulations demonstrated significant effects on this parameter.

**Figure 2:**
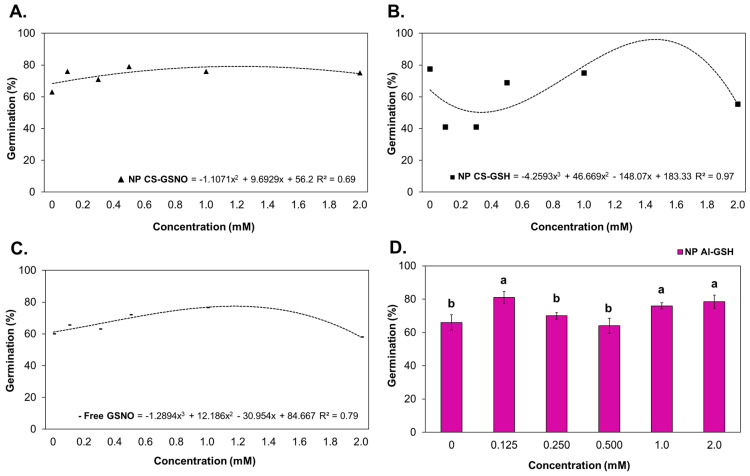
Germination rate (%) of *Glycine max* (L.) Merr. seeds following treatment with chitosan nanoparticles with and without NO (NP CS-GSNO and NP CS-GSH), alginate nanoparticles without NO (NP Al-GSH), and free GSNO. (**A**–**C**) Regression curves were fitted for concentrations within each formulation (*p* < 0.05), namely NP CS-GSNO, NP CS-GSH, and free GSNO, respectively. The values shown represent the mean of four replicates. (**D**) Different letters above the bars indicate different means based on ANOVA followed by the Scott–Knott test for the NP Al-GSH treatment (*p* ≤ 0.05). Data are expressed as mean ± standard error (n = 4).

Regression curves were constructed for the NP CS-GSNO, NP CS-GSH, and GSNO treatments in order to ascertain the germination percentage (Figure 2). The seed treatment with NP CS-GSNO 1.0 mM demonstrated a positive effect when compared with the control, resulting in an increased germination rate (71%) compared with the control (60%). Similar effects were observed for other concentrations. At 0.125 and 0.250 mM, NP CS-GSH induced a reduction in germination compared with the control, with decreases of 49% and 41%, respectively. A concentration of 2.0 mM resulted in a reduction in germination compared with the control for both the NP CS-GSH and free GSNO formulations. For the NP Al-GSH formulation, the data were compared using the Scott–Knott test (Figure 2D), as no regression curves were fitted. The optimal concentrations were identified as 0.125, 1.0, and 2.0 mM, exhibiting increases of 16%, 11%, and 14%, respectively, in comparison with the control. It is noteworthy that, in contrast with NP CS-GSH, the lowest concentration did not result in a depression of the germination rate.

**Figure 3:**
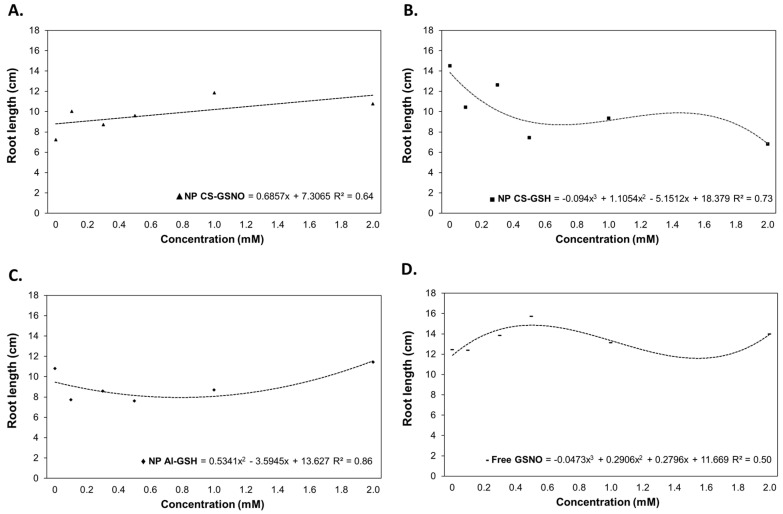
Root length (cm) of *Glycine max* (L.) Merr. seedlings following seed treatment with chitosan nanoparticles with and without NO (NP CS-GSNO and NP CS-GSH), alginate nanoparticles without NO (NP Al-GSH), and free GSNO. The control treatment (zero concentration) refers to seeds treated with distilled water only. The values shown represent the mean of four replicates. Regression curves were fitted for concentrations within each formulation (*p* ≤ 0.05).

Regarding the root length (RL), regression curves were constructed for the treatments with NP CS-GSNO, NP CS-GSH, NP Al-GSH, and free GSNO (Figure 3). The ANOVA was not statistically significant only for NP Al-GSNO (Table S1). The NP CS-GSH treatment exhibited a deleterious effect on RL in comparison with the control. At 0.125, 0.250, and 0.500 mM, the NP Al-GSH formulation also resulted in a reduction in this parameter. In contrast, the NP CS-GSNO and free GSNO treatments were observed to increase RL. The highest RL for NP CS-GSNO was observed at 1.0 mM with an increasing trend, while the highest RL for free GSNO was observed at 0.500 mM, followed by a decreasing trend.

**Figure 4:**
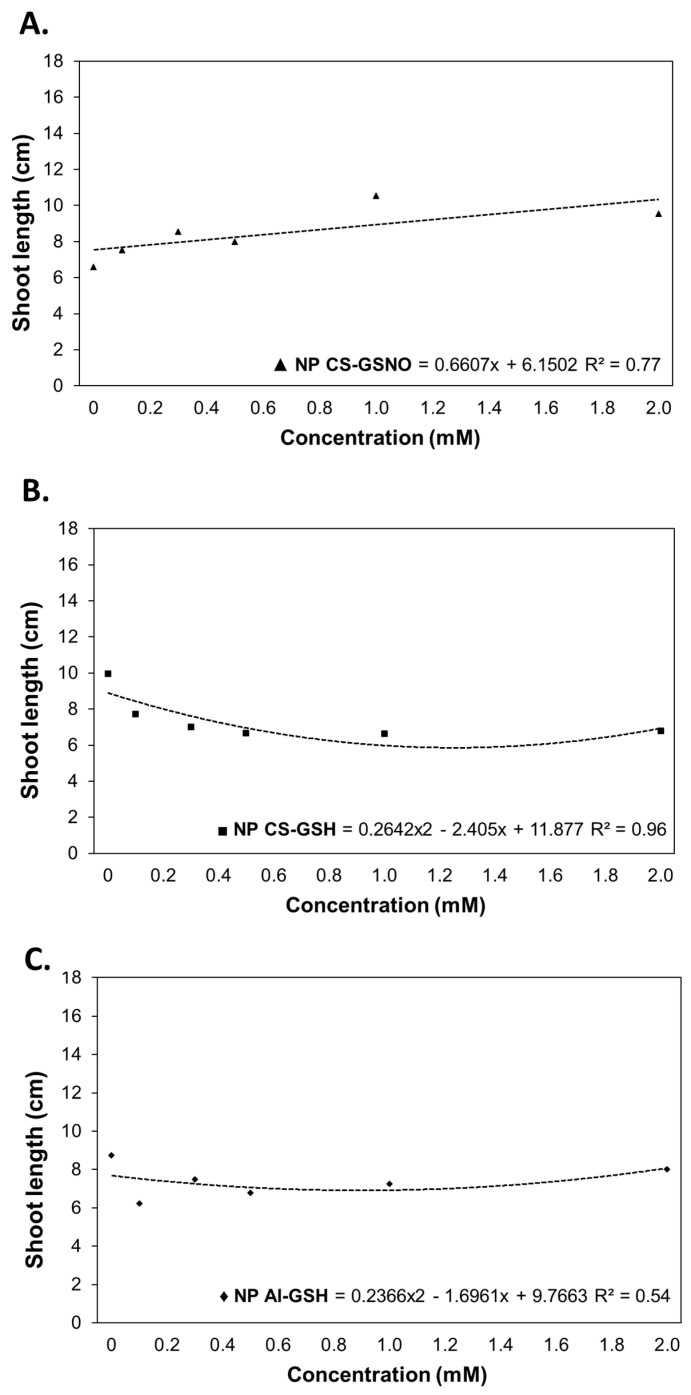
Shoot length (cm) of *Glycine max* (L.) Merr. seedlings following seed treatment with chitosan nanoparticles with and without NO (NP CS-GSNO and NP CS-GSH) and alginate nanoparticles without NO (NP Al-GSH). The control treatment (zero concentration) refers to seeds treated with distilled water only. The values shown represent the mean (n = 4). Regression curves were fitted for concentrations within each formulation (*p* ≤ 0.05).

For shoot length (SL), regression curves were constructed for the treatments NP CS-GSNO, NP CS-GSH, and NP Al-GSH (Figure 4). The results of the ANOVA for NP Al-GSNO and free GSNO were not statistically significant (Table S1). The NP CS-GSH and NP Al-GSH treatments exhibited a deleterious impact on this parameter, with reductions of approximately 54% at a concentration of 2.0 mM and 28% at intermediate concentrations (0.250 and 0.500 mM), respectively, in comparison with the control. In contrast, seeds treated with NP CS-GSNO demonstrated a gradual increase in shoot length.

**Figure 5:**
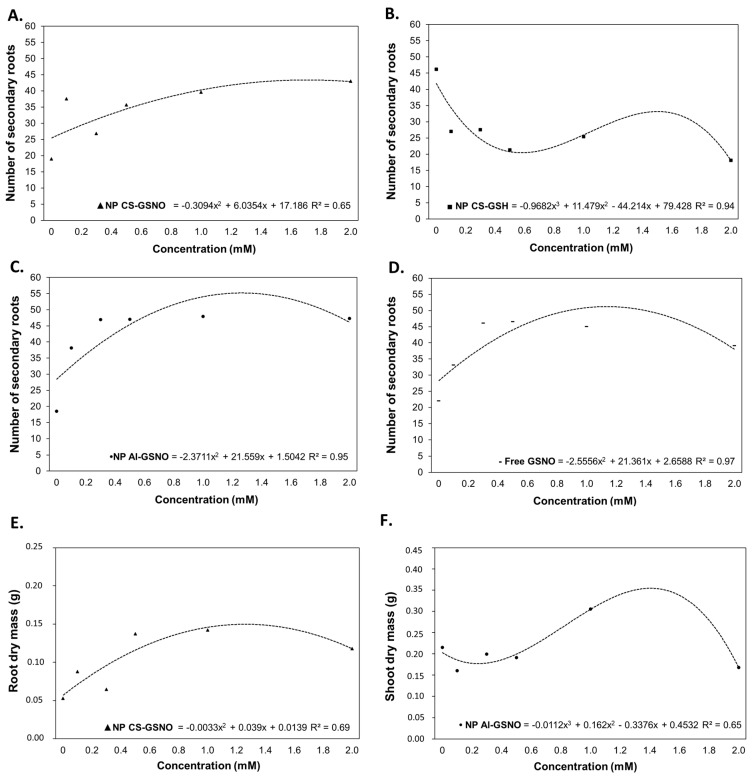
Number of secondary roots (**A**–**D**), root dry weight (**E**), and shoot dry weight (**F**) of *Glycine max* (L.) Merr seedlings following seed treatment with chitosan nanoparticles with and without NO (NP CS-GSNO and NP CS-GSH), alginate nanoparticles with NO (NP Al-GSNO), and free GSNO. The control treatment (zero) refers to seeds treated with distilled water only. The values shown represent the mean of four replicates. Regression curves were fitted for concentrations within each formulation, and the resulting models were statistically significant (*p* ≤ 0.05).

For the number of secondary roots (NSR), regression curves were fitted for the treatments with NP CS-GSNO, NP Al-GSNO, NP CS-GSH, and free GSNO (Figure 5). The ANOVA was not statistically significant only for NP Al-GSH (Table S1). As with RL, NP CS-GSH was the only treatment that demonstrated a reduction in this parameter compared with the control. The optimal concentration for NP CS-GSNO was determined to be 2.0 mM, while for NP Al-GSNO it was 0.250 mM, with similar values observed at other concentrations. In general, NP Al-GSNO induced NSR values approximately threefold higher than the control. The effect of free GSNO on NSR was similar to that of NP Al-GSNO, but at the highest concentration there was a slight decrease.

The NP CS-GSNO formulation was the only one to yield a statistically significant regression curve for the root dry mass (RDM) (Figure 5E and Table S1). It is noteworthy that an increase in RDM was observed at higher concentrations of NP CS-GSNO. For the shoot dry mass (SDM), regression fitting was feasible only for NP Al-GSNO (Figure 5F and Table S1), which resulted in an increase of approximately 37% at 1 mM in comparison with the control.

The findings of these in vitro experiments allowed us to establish 1.0 mM as the optimal dose of GSNO/GSH for the subsequent experiments.

#### 2.2.2. Imbibition Curve

**Figure 6:**
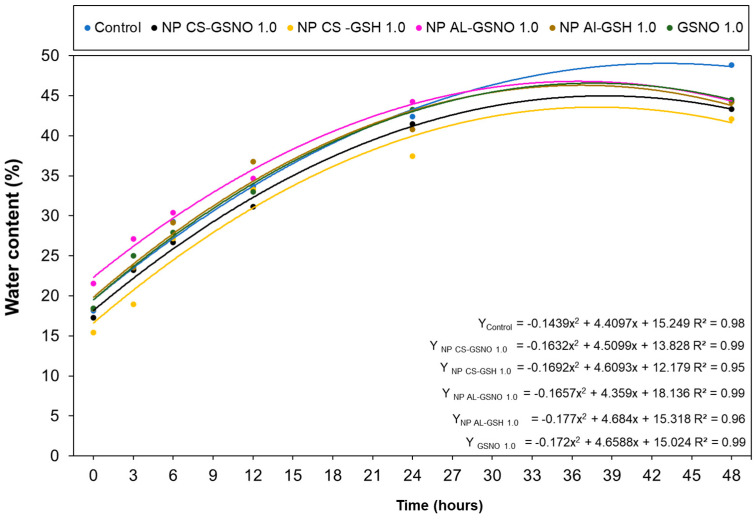
Water content (%) in *Glycine max* (L.) Merr. seeds at increasing time intervals following treatment with chitosan nanoparticles with and without NO (NP CS-GSNO and NP CS-GSH), alginate nanoparticles with and without NO (NP Al-GSNO and NP Al-GSH), free GSNO, and water (control). A concentration of 1.0 mM of GSNO/GSH was used in this experiment. The values shown represent the mean of four replicates. A regression curve was fitted for each treatment group over time (*p* ≤ 0.05).

We also evaluated the impact of the seed treatments with different formulations at 1 mM on the water uptake by the seeds. The initial water content of the seeds was approximately 8% (value obtained using a G650i (Gehaka Serial RS232C, Gehaka, São Paulo, Brazil) moisture and impurity analyzer). Immediately following the treatments, the seed water content values increased to 18%, 17%, 16%, 22%, 18%, and 19% for the control, NP CS-GSNO, NP CS-GSH, NP Al-GSNO, NP Al-GSH, and free GSNO, respectively (Figure 6). The absorption exhibited a rapid initial increase, followed by gradual stabilization, regardless of the treatment. However, when comparing the NO-releasing nanoparticles, NP Al-GSNO exhibited a higher water content in the first 24 h, which might indicate a superior absorption capacity following treatment or greater efficiency in the initial metabolic reactivation. At 48 h, the highest water content (48%) was observed in the control seeds (soaked only in water).

#### 2.2.3. Biochemical Analyses

**Figure 7:**
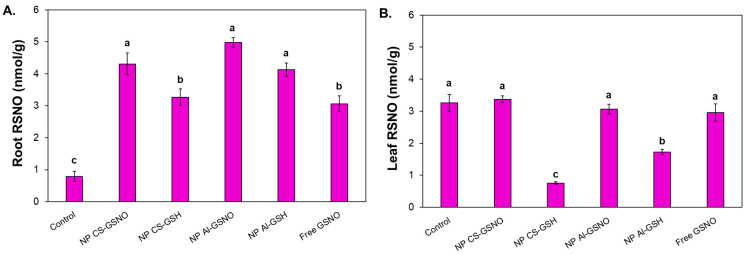
Effect of seed treatment with chitosan nanoparticles with and without NO (NP CS-GSNO and NP CS-GSH), alginate nanoparticles with and without NO (NP Al-GSNO and NP Al-GSH), free GSNO, and distilled water (control) on the S-nitrosothiol (RSNO) content in roots (**A**) and leaves (**B**) of soybean seedlings collected after 8 days. A concentration of 1.0 mM of GSNO/GSH was used in this experiment. The values are the mean ± standard error (n = 4). The presence of different letters above the bars indicates that the means are significantly different, as determined by ANOVA followed by the Scott–Knott test (*p* ≤ 0.05).

In the roots, the highest RSNO levels were observed in the NP CS-GSNO, NP Al-GSNO, and NP Al-GSH treatments (approximately fourfold higher compared with the control) (Figure 7A). A noteworthy reduction in RSNO concentration was observed in the leaves of seedlings from seeds that received NP CS-GSH and NP Al-GSH treatments (Figure 7B). The leaf RSNO levels in the NP CS-GSNO, NP Al-GSNO, and GSNO treatments were comparable to those of the control.

### 2.3. Greenhouse Experiment

Seeds of the control and those treated with the formulations at 1 mM GSNO/GSH were sown in greenhouse conditions to evaluate the long-term effects of the treatments on soybean plants. The results of the ANOVA are presented in Table S1.

**Figure 8:**
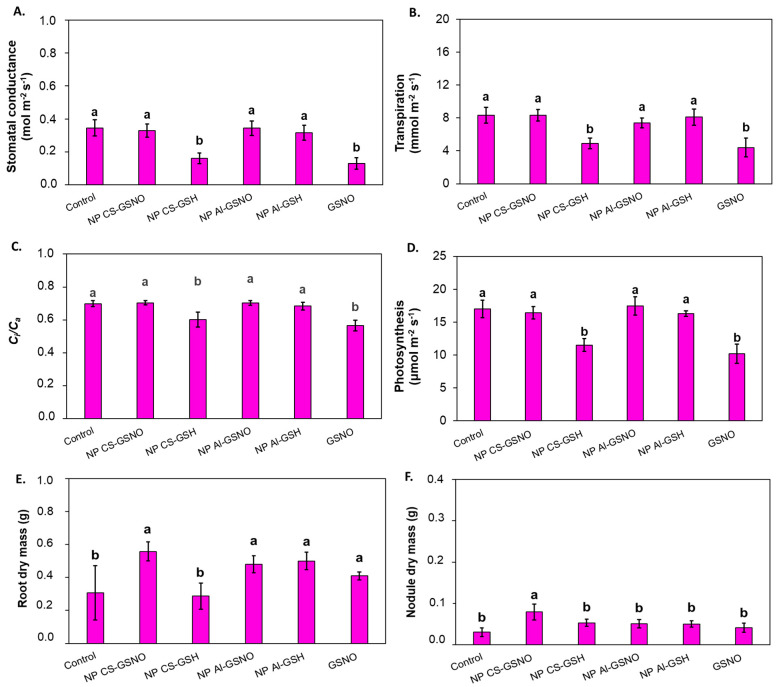
Effect of seed treatment with chitosan nanoparticles with and without NO (NP CS-GSNO and NP CS-GSH), alginate nanoparticles with and without NO (NP Al-GSNO and NP Al-GSH), free GSNO, and water (control) on (**A**) stomatal conductance (mol m^−2^ s^−1^), (**B**) transpiration (mmol m^−2^ s^−1^), (**C**) internal/ambient CO₂ concentration (*C*_i_/*C*_a_), (**D**) net photosynthesis (μmol m^−2^ s^−1^), (**E**) root dry mass, and (**F**) nodule dry mass of soybean plants after 35 days of growth in a greenhouse. The values are the mean ± standard error (n = 6). Columns with different letters indicate statistically significant differences (*p* ≤ 0.05) based on ANOVA followed by the Scott–Knott test.

Stomatal conductance was twofold lower in the NP CS-GSH and free GSNO treatments in comparison with the control and other formulations (Figure 8). A comparable outcome was observed with respect to transpiration rate, *C*_i_/*C*_a_, and net photosynthetic rate. In contrast, the NP CS-GSNO, NP Al-GSNO, and NP Al-GSH treatments did not affect the leaf gas exchange parameters compared with the control.

Statistically significant treatment effects were observed for root dry mass (RDM) and nodule dry mass (NDM) (Figure 8E,F). The seed treatments with NP CS-GSNO, NP Al-GSNO, NP Al GSH, and free GSNO induced an increase in RDM compared with the control and NP CS-GSH treatment. Plants from the NP CS-GSNO treatment also exhibited a higher NDM compared with the other treatments.

The fresh mass parameters (shoot, root, and nodules), total number of nodules, length of shoots and roots, and shoot dry mass did not differ significantly between treatments (Table S1, Figure S2).

## 3. Discussion

Several studies have demonstrated that the effects of nanoparticles may depend on their physicochemical properties (including size, chemical composition, and surface charge), the tested plant species, and application methods [14,21,26,27,28,29,30] In the present study, we tested the effect of the seed treatment with NO-releasing chitosan and alginate nanoparticles on the germination and initial development of soybean plants. These nanoparticles show opposite surface charges (positive in chitosan and negative in alginate). In addition, chitosan nanoparticles have a larger hydrodynamic diameter and a faster NO release profile compared with alginate nanoparticles.

Overall, we observed that NP CS-GSNO induced more positive effects on soybean plants than NP Al-GSNO. For example, in the in vitro assay, seed treatment with NP CS-GSNO promoted dose-dependent increases in germination percentage, RL, SL, NSR, and RDM, while the treatment with NP Al-GSNO stimulated only the NSR and SDM. The positive charge of NP CS-GSNO probably favored adhesion to the seed coat, resulting in a more gradual release of the nanoencapsulated compound over time [24,25,31]. On the other hand, the negative charge of NP Al-GSNO might have facilitated its internalization into the seeds. Accordingly, this nanoformulation resulted in a more immediate effect on the seeds, particularly as observed in the water absorption test in the first 24 h.

Treatments with free GSNO and NPs containing GSNO demonstrated important differences regarding the initial development of soybean plants, highlighting the benefits of nanoencapsulation. While seed treatment with free GSNO demonstrated positive effects on germination and RL, NP CS-GSNO, especially at the 1 mM concentration, provided broader benefits, as listed above. Our results so far indicate that the beneficial effects observed in GSNO NP treatments are attributable to the NO donor and/or the controlled release of NO, since NPs containing non-nitrosated GSH showed less favorable responses in the initial development of the plants. The benefits of nanoencapsulation of NO donor molecules have already been demonstrated in the literature in several plant species, including soybean, sugarcane (*Saccharum officinarum*), and neotropical tree seedlings [32,33,34].

NO integrates multiple signaling pathways, interacting with Ca^2+^, GSH, reactive oxygen species (ROS), and plant hormones. These interactions are of great importance for the regulation of several physiological processes, particularly during the germination phase. For example, NO plays a regulatory role in Ca^2+^ homeostasis, which is essential for cellular signaling and enzyme activation during germination. By interacting with GSH, NO participates in the mitigation of oxidative stress, thereby protecting seeds from potential damage during this phase [12,35,36,37]. The integration of NO with plant hormones such as ABA and GA is also of great importance, as these hormones have opposing functions in germination; ABA tends to inhibit germination, while GA promotes it [38]. Therefore, NO plays a crucial role in modulating these hormones, creating a cellular environment conducive to metabolic activation and the early development of seedlings.

Steven et al. (2024) observed that the germination rates of soybean seeds increased by 20.3, 12.8, and 7.7% after 3, 4, and 5 h of seed treatment with NP CS-GSNO, respectively, in comparison with the control. Similarly, we observed increased germination rates for the NP CS-GSNO treatment at all concentrations, even with a shorter exposure period (5 min). The authors also observed an increase in GA levels and a decrease in ABA levels in response to NP CS-GSNO [22]. These hormonal alterations may elucidate how NO can modulate the germination process, thereby creating a more conducive environment for the initial stages of germination.

Another noteworthy finding was the increased content of RSNOs in the roots of plants that received the seed treatment with nanoencapsulated GSNO, which allows us to infer greater NO availability [39]. Furthermore, we observed an increase in the number of secondary roots for NP CS-GSNO, NP Al-GSNO, and GSNO treatments, which highlights the crucial role of NO in regulating root architecture. NO has been demonstrated to modulate the signaling of hormones involved in root growth and development, with the potential to promote increased primary root length, lateral root formation, and root hair density. In particular, NO interacts synergistically with auxin, often amplifying the effects of this molecule on cell expansion and root morphogenesis. However, these outcomes can vary based on NO concentration, environmental conditions, and exposure time, highlighting the complexity of NO–auxin interactions in plant development [40]. Additionally, NO plays a role in nearly all stages of the legume–rhizobium symbiosis process [13,39,41,42,43].

Given the promising results observed during the in vitro studies, our research proceeded to assess the efficacy of the seed treatments in a greenhouse environment, by evaluating their impact on plant morphophysiological attributes, including the root nodulation. As indicated in the literature, an increase in NO production occurs during the early stages of the legume–rhizobium interaction, specifically in the root branching zones. Furthermore, NO has been detected during the initial formation, mature phase, and senescence of nodules, indicating roles in both signaling and metabolic functions of the symbiotic process [42,44,45].

Regarding the physiological parameters, the stomatal conductance was lower in plants of the NP CS-GSH and free GSNO treatments in comparison with the control. This reduced stomatal conductance was related to lower transpiration and lower CO_2_ levels in the leaves, leading to impaired photosynthesis. In contrast, the NP CS-GSNO, NP Al-GSNO, and NP Al-GSH treatments maintained these leaf gas exchange parameters comparable to the control. Therefore, GSNO nanoencapsulation prevented the negative effects of the NO donor on leaf gas exchange.

Prior research has indicated that NO is crucial for regulating stomatal function. However, NO can have either a positive or negative effect on stomatal conductance, depending on its concentration. [34,45,46]. As previously discussed, NO is a key mediator in the ABA signaling pathway, regulating stomatal movement [47]. Evidence suggests that NO production via the NR-mediated pathway activates a mitogen-activated protein kinase (MAPK) cascade, leading to stomatal closure [19,47,48,49,50]. This mechanism may explain the observed reduction in stomatal conductance and the subsequent decrease in photosynthesis of plants from the GSNO treatment, suggesting a potential long-term inhibitory effect on photosynthetic performance.

An important long-term effect of NP CS-GSNO treatment was the increase in nodule dry mass, which is in accordance with the beneficial effects of this treatment observed during the in vitro experiments. Calvo-Begueira et al. (2018) [51] identified the formation of NO in infected cells of soybean and bean (*Phaseolus vulgaris*) nodules, and in the parenchyma of soybean nodules. In addition, Methela et al. (2023) [32] observed that the seed treatment with GSNO-loaded chitosan nanoparticles significantly enhanced photosynthetic parameters and the number of nodules in soybean plants. Herein, we also observed a positive effect of the seed treatment with NP CS-GSNO on the growth of soybean roots, an effect that was also induced by the other NO-releasing formulations.

## 4. Conclusions

Our findings highlight the potential of combining nanoparticles and NO donors in seed treatment as an effective approach to enhance plant growth and development. Both chitosan and alginate nanoparticles yielded promising results in in vitro and greenhouse conditions, indicating the versatility of nanoencapsulation in delivering NO to plants. Among the treatments, NP CS-GSNO at a concentration of 1.0 mM of the NO donor was identified as the most effective, improving germination rate, root architecture, root RSNO content, and nodule growth of soybean plants. Although alginate nanoparticles with GSNO induced faster water absorption during seed treatment, chitosan nanoparticles demonstrated superior long-term effects on plant development. The observed diversity of effects underscores the impact of the physicochemical properties of nanoparticles and their interactions with diverse plant species. As such, further studies are necessary to deepen our understanding of these interactions and to optimize the design and application of NO-releasing nanoparticles for agricultural purposes.

Overall, this research supports the use of nanoencapsulated NO donors as a promising strategy to improve germination, enhance root development, and foster beneficial symbiotic relationships in crops, offering significant potential for advancing sustainable agricultural practices.

## 5. Material and Methods

### 5.1. Preparation of Formulations

#### 5.1.1. Chitosan Nanoparticles

Chitosan nanoparticles (NPs CS) were prepared through ionotropic gelation, as previously described by Silveira et al. (2019) [34] and Seabra et al. (2017) [52]. In brief, chitosan (2.6 mg mL^−1^) and reduced glutathione (GSH) (20 mM) were mixed by magnetic stirring in an aqueous solution of acetic acid (1%) for 90 min. Sodium tripolyphosphate (TPP) (0.6 mg mL^−1^) was added dropwise to the chitosan/GSH suspension, maintaining a volumetric ratio of 3 CS/GSH:1 TPP. The mixture was stirred for 90 min at room temperature, resulting in the formation of an aqueous suspension of NPs CS containing GSH (without NO) at 20 mM. To obtain the NPs CS containing GSNO, a S-nitrosation reaction of GSH was performed by adding equimolar amounts of sodium nitrite (NaNO_2_) to the NPs CS-GSH suspension. The final suspension was homogenized and kept in the dark for 60 min at 5 °C [47]. Treatments with NPs CS containing non-S-nitrosated GSH were also employed to assess the impact of the formulation without NO.

#### 5.1.2. Alginate Nanoparticles

Alginate nanoparticles (NPs Al) were synthesized through ionotropic gelation with calcium ions (Ca^2+^). An aqueous solution (40 mL) of alginate (0.475 mg mL^−1^) was prepared and stirred for a period of three hours. The alginate and GSH were combined via magnetic stirring for a period of five minutes. Subsequently, the required quantity of GSH was dissolved in conjunction with 6 mL of the alginate solution. A peristaltic pump (Marte MVA-500, Marte Científica, Santa Rita do Sapucaí, Brazil) was employed to introduce 4.0 mL of CaCl_2_ (2.25 mmol L^−1^) to the initial solution at a rate of 2 drops per minute. Subsequently, the mixture was stirred for one hour, resulting in the formation of NPs Al containing GSH [53]. To obtain alginate nanoparticles containing GSNO, S-nitrosation reaction of GSH was performed by adding equimolar amounts of NaNO_2_ to the GSH-containing NPs Al suspension. The final suspension was stirred for 60 min at 5 °C, protected from light, thereby forming NPs Al-GSNO [52]. Furthermore, treatments with NPs Al containing non-S-nitrosated GSH were conducted to evaluate the influence of the formulation lacking NO.

#### 5.1.3. Free S-Nitrosoglutathione

To obtain the free NO donor GSNO, GSH was dissolved in hydrochloric acid (1 mol L^−1^). An equimolar quantity of NaNO_2_ was introduced to the GSH solution and maintained in an ice bath for 40 min, with magnetic stirring. Subsequently, acetone was added to facilitate the precipitation of GSNO. The resulting precipitate of GSNO was then subjected to multiple washes with cold water, and the solid was subsequently lyophilized for 48 h [54].

### 5.2. Characterization of Nanoparticles and NO Release

Dynamic light scattering (DLS) (Nano ZS Zetasizer, Malvern Inst. Ltd., Worcestershire, UK) was employed to determine the hydrodynamic size of the nanoparticles (by intensity), the polydispersity index (PDI), and the zeta potential [26,33,34,55]. The NO release profile of nanoencapsulated formulations (both at 20 mM of GSNO) was monitored by ultraviolet (UV) spectroscopy through spectral changes at 545 nm [26,56,57]. Absorbance measurements were taken at 30 min intervals for the nanoencapsulated samples, which were maintained at room temperature (25 ± 1 °C) for a period of 5 h. The data points on the kinetic curve represent the mean of three independent experiments in triplicates, with the data expressed as the total rate of NO released.

### 5.3. Plant Material and Treatments

Soybean seeds [*Glycine max* (L.) Merr.] from a conventional cultivar (BRS 257) were provided by Embrapa Soja Londrina. This cultivar was developed specifically for human consumption, as the variety is free of the lipoxygenase enzymes responsible for giving the products their bitter taste. It has a determined growth habit, a short white flower maturation cycle, and a light brown hilum [58].

#### 5.3.1. In Vitro Experiment

The following treatments were utilized in the in vitro assays: (a) control (distilled water); (b) NPs CS containing GSNO (NP CS-GSNO); (c) NPs Al containing GSNO (NP Al-GSNO); (d) NPs CS-GSH (without NO); (e) NPs Al-GSH (without NO); and (f) free GSNO. The tested concentrations of GSNO or GSH were 0.125 mM, 0.250 mM, 0.500 mM, 1.0 mM, and 2.0 mM.

Tests were carried out to determine the maximum duration of seed soaking that did not cause imbibition damage. For this, seeds were immersed in water for 1, 5, 10, or 15 min. At 10 and 15 min of soaking, damage was observed in more than 50% of the seeds tested, indicating adverse effects of imbibition. In contrast, at 1 and 5 min, no discernible difference in germination percentage was observed in comparison with non-imbibed seeds (results not shown). Thus, a five-minute soaking time was used for the seed treatment with the formulations.

For each treatment, 4 biological replicates were used, each comprising 50 seeds (a total of 200 seeds per treatment). The seeds were placed in beakers containing 100 mL of the aforementioned formulations and were kept under agitation for five minutes. Subsequently, the seeds were subjected to a drying process at room temperature. Afterwards, they were positioned on moistened Germitest paper, with an amount of distilled water equivalent to 2.5 times the mass of the dry paper. The paper rolls were then placed in a BOD incubator for 8 days at 25 °C with a 12 h photoperiod (Figure S3, Steps 1 and 2).

##### Germination Analyses

The germination percentage was assessed on the eighth day after sowing, using the formula delineated in Equation (1).

(1) Germination %=NTS×100

where *N* represents the number of germinated seeds, and *TS* represents the total number of seeds utilized.

A seedling was considered to be normal when it exhibited a protruding radicle exceeding 1 mm in length and displayed typical development of the mesocotyl [59,60]. Shoot length (SL), root length (RL), number of secondary roots (NSR), shoot dry mass (SDM), and root dry mass (RDM) were evaluated in 10 randomly selected seedlings per replicate. To determine the dry mass, the samples were maintained in an oven at 70 °C for 72 h.

##### Water Absorption Assay

Plastic 11 × 11 cm gerbox containers, each containing 50 seeds, were employed in the experiment. The filter paper that was holding the seeds was moistened with distilled water at a ratio of two-and-half times the weight of the dry paper. Immediately following the completion of the treatment, the seeds were meticulously dried and weighed to obtain the initial fresh mass (FM). The seeds were then transferred to a gerbox and weighed at 3 h intervals over a 48 h period. Subsequently, the seeds were dried in an oven at 70 °C to determine the dry mass (DM). The water content of the seeds at each time point was calculated using the following equation, as proposed by Preisler et al. (2022) and Oliveira and Bosco (2013) [25,61].

(2) $$Water\;content(\%)=\left(\frac{FM-DM}{FM}\right)\times 100$$

##### Biochemical Analysis

On the eighth day of the in vitro assay, samples were collected (100 mg), ground to a powder in liquid nitrogen, and stored at −80 °C. The content of S-nitrosothiols (RSNOs) in the roots and shoots was obtained as an indicator of NO bioavailability. The samples were mixed with 1 mL of NEM solution (5 mmol L^−1^ prepared in PBS, pH 7.4), sonicated for 10 s (45 kHz), and centrifuged for 5 min at 15,645 × g. Solutions of 0.1 mol L^−1^ CuCl_2_ were prepared and used to stabilize the sensor. The data were collected using a free radical analyzer (WPI TBR4100/1025, World Precision Instruments Inc., Sarasota, FL, USA) equipped with a specific NO sensor (ISO-NOP 2 mm), to which 20 μL of plant extract was added. Subsequently, the data were compared with the standard curve obtained for GSNO. Analyses were conducted in triplicate for each sample, as previously described [26,34,55].

#### 5.3.2. Greenhouse Experiment

The greenhouse experiment was conducted using only the formulations at 1.0 mM of GSNO/GSH. Each treatment was conducted with 6 replicates. The seeds were treated for five minutes in beakers containing 100 mL of the formulations or water as the control, in accordance with the previously outlined methodology (Figure S3, Steps 1 and *3*). Subsequently, the seeds were inoculated individually with a commercial inoculant based on *Bradyrhizobium japonicum* (7 × 10^9^ CFU mL^−1^), in accordance with the manufacturer’s technical recommendations.

Plastic pots (10.5 cm height, 14.5 cm top diameter, 1 L total volume) were filled with approximately 800 g of red Latosol, which was collected from an actively cultivated area. Three seeds were sown per pot and, after 14 days, only one plant per pot was retained for the experiment. Following a cultivation period of 35 days, the soybean plants reached the V4 stage and their physiological and morphological parameters were evaluated.

The youngest fully expanded leaf of each plant was utilized for the gas exchange analysis, which was carried out on a sunny day between 7 a.m. and 11 a.m. The following variables were determined using a portable infrared gas analyzer (model LICOR 6400 XT, Biosciences, Lincoln, NE, USA): net photosynthesis, stomatal conductance, transpiration, and the ratio of internal to ambient CO_2_ concentrations (*C*_i_/*C*_a_). The apparatus was connected to a 6 cm^2^ chamber under saturating photosynthetically active radiation (1500 μmol m^−2^ s^−1^), with a flow rate of 400 mL min^−1^.

The morphological parameters of each plant were analyzed, including shoot and root length, number of nodules, and dry mass of shoots, roots, and nodules. To determine the dry mass, the plant material was subjected to an oven-drying process at 70 °C for 72 h.

### 5.4. Statistical Analyses

A series of tests were conducted on the data to ascertain their suitability for further analysis. These included a Shapiro–Wilk W test to confirm the normality of the data set and a Bartlett’s test to assess the homogeneity of variances. A one-way analysis of variance (ANOVA) was conducted for each parameter (results presented in Table S1). In the case of germination assays, the data were subjected to polynomial regression modeling. In cases where fitting was not possible, the means were compared using the Scott–Knott test. In the case of biochemical analyses and greenhouse assays, the Scott–Knott test was also employed for the comparison of significant data. In order to validate the effect of the various treatments on water absorption, one-way ANOVA was employed to fit equations for time in each treatment. All analyses were carried out with a significance level of *p* ≤ 0.05, considering two decimal places.

## Data Availability

Data supporting reported results can be found at https://docs.google.com/spreadsheets/d/1gDTnK3LUkiUxxbMMC7y5ANH79f6IIWQL (accessed on 16 December 2024).

## Supplementary Materials

The following supporting information can be downloaded at: https://www.mdpi.com/article/10.3390/plants14010017/s1. Table S1 Analysis of variance, *p* values and regression models. Asterisks indicate significance according to ANOVA (*p* ≤ 0.05). ^ns^ = non-significant. Figure S1. Representative images of *Glycine max* (L.) Merr. seedlings from the treatments: nanoparticles with chitosan nanoparticles with and without NO (NP CS-GSNO and NP CS-GSH), alginate nanoparticles with NO (NP Al-GSNO), free GSNO, and control (water). Concentrations of 0.125 mM, 0.250 mM, 0.500 mM, 1.0 mM, and 2.0 mM of GSNO/GSH were used. Figure S2. Effect of seed treatment with chitosan nanoparticles with and without NO (NP CS-GSNO and NP CS-GSH), alginate nanoparticles with and without NO (NP Al-GSNO and NP Al-GSH), and GSNO on plant growth. A concentration of 1.0 mM of GSNO/GSH was used in this experiment. The control treatment refers to seeds treated with distilled water only. The following variables were measured for *Glycine max* (L.) Merr. plants grown in a greenhouse: fresh mass (A), total nodule number (B), length (C), and shoot dry mass (D). The values presented represent the mean of six replicates ± the standard error. No significant difference was detected based on the results of the ANOVA followed by the Scott-Knott test (*p* ≤ 0.05). Figure S3. Representative scheme of the treatments of the seeds. *Step 1* refers to the nitrosation of nanoparticles with sodium nitrite (NaNO_2_) and the seed treatment by immersion. *Step 2* covers the procedures conducted during germination and early seedling growth experiments. *Step 3* involves the procedures carried out during the nodulation experiment.
