# Supplementary material for: Improving Soybean Germination and Nodule Development with Nitric Oxide-Releasing Polymeric Nanoparticles
Source: Plants (Basel). 2024 Dec 25;14(1):17. doi: 10.3390/plants14010017 (PMC11723237; doi:10.3390/plants14010017)
Supplement: Supplementary file 1 [file plants-14-00017-s001.zip › plants-3369525-supplementary.pdf]

*Article*

# Improving Soybean Germination and Nodule Development with Nitric Oxide-Releasing Polymeric Nanoparticles

**Ana Cristina Preisler [1,2], Giovanna Camargo do Carmo [1], Rafael Caetano da Silva [3], Ana Luisa de Oliveira Simões [1,2], Juliana de Carvalho Izidoro [4], Joana Claudio Pieretti [4], Roberta Albino dos Reis [4], André Luiz Floriano Jacob [4], Amedea Barozzi Seabra [4] and Halley Caixeta Oliveira [1,*]**

1 Department of Animal and Plant Biology, Londrina State University, Londrina 86057-970, PR, Brazil; preislerac@gmail.com (A.C.P.); giovannacdcarmo@gmail.com (G.C.d.C.); ana.luisa.oliveira@uel.br (A.L.d.O.S.)
2 Department of Agronomy, Londrina State University, Londrina 86057-970, PR, Brazil
3 Department of Biodiversity Conservation, Institute of Environmental Research, São Paulo 04301-902, SP, Brazil; rafael.caetano94@gmail.com
4 Center for Natural and Human Sciences (CCNH), Federal University of ABC (UFABC), Santo André 09210-580, SP, Brazil; julianaizidoro@yahoo.com.br (J.d.C.I.); joana.pieretti@ufabc.edu.br (J.C.P.); roberta.reis@ufabc.edu.br (R.A.d.R.); andre.jacob@aluno.ufabc.edu.br (A.L.F.J.); amedea.seabra@ufabc.edu.br (A.B.S.)
* Correspondence: halley@uel.br

SUPPLEMENTARY INFORMATION

**Table S1** Analysis of variance, p values and regression models. Asterisks indicate significance according to ANOVA ($p \leq 0.05$). [ns] = non-significant.

| Treatment | Figure number | F value | ANOVA | Normality test | Homogeneity test | Adjusted regression model | $R^2$ |
|---|---|---|---|---|---|---|---|
| NP CS-GSNO | Fig 2 A. | 5.474 | 0.00* | 0.8107 | 0.6157 | $y = -1.1071x^2 + 9.6929x + 56.2$ | 0.69 |
| NP CS-GSH | Fig 2 B. | 6.319 | 0.00* | 0.4437 | 0.2489 | $y = -4.2593x^3 + 46.669x^2 - 148.07x + 183.33$ | 0.97 |
| Free GSNO | Fig 2 C. | 1.675 | 0.05* | 0.1656 | 0.1722 | $y = -1.2894x^3 + 12.186x^2 - 30.954x + 84.667$ | 0.79 |
| NP Al-GSH | Fig 2 D. | 2.995 | 0.03* | 0.0996 | 0.5808 | not adjusted | - |
| NP Al-GSNO | - | 1.592 | 0.21[ns] | 0.1428 | 0.4277 | - | - |
| | | | | | | | |
| NP CS-GSNO | Fig 3 A. | 2.645 | 0.05* | 0.5847 | 0.1800 | $y = 0.6857x + 7.3065$ | 0.64 |
| NP CS-GSH | Fig 3 B. | 10.451 | 0.00* | 0.2970 | 0.0912 | $y = -0.094x^3 + 1.1054x^2 - 5.1512x + 18.379$ | 0.73 |
| NP Al-GSH | Fig 3 C. | 2.854 | 0.04* | 0.2532 | 0.6054 | $y = 0.5341x^2 - 3.5945x + 13.627$ | 0.86 |
| Free GSNO | Fig 3 D. | 3.400 | 0.02* | 0.7999 | 0.8920 | $y = -0.0473x^3 + 0.2906x^2 + 0.2796x + 11.669$ | 0.50 |

| | | | | | | | | |
|---|---|---|---|---|---|---|---|---|
| NP Al-GSNO | | - | 1.970 | $0.13^{ns}$ | 0.5140 | 0.3924 | - | - |
| | | | | | | | | |
| NP CS-GSNO | | Fig 4 A. | 3.575 | 0.02* | 0.9209 | 0.1883 | y = 0.6607x + 6.1502 | 0.77 |
| NP CS-GSH | | Fig 4 B. | 8.780 | 0.00* | 0.6085 | 0.5049 | y = 0.2642x2 - 2.405x + 11.877 | 0.96 |
| NP Al-GSH | | Fig 4 C. | 2.898 | 0.04* | 0.0954 | 0.5708 | y = 0.2366x2 - 1.6961x + 9.7663 | 0.54 |
| Free GSNO | | - | 1.477 | $0.24^{ns}$ | 0.8615 | 0.4123 | - | - |
| NP Al-GSNO | | - | 2.103 | $0.11^{ns}$ | 0.9558 | 0.2952 | - | - |
| | | | | | | | | |
| NP CS-GSNO | | Fig 5 A. | 8.353 | 0.00* | 0.8107 | 0.6157 | $y = -0.3094x^2 + 6.0354x + 17.186$ | 0.65 |
| NP CS-GSH | | Fig 5 B. | 18.336 | 0.00* | 0.6040 | 0.3544 | $y = -0.9682x^3 + 11.479x^2 - 44.214x + 79.428$ | 0.94 |
| NP Al-GSNO | | Fig 5 C. | 2.804 | 0.04* | 0.5561 | 0.0912 | $y = -2.3711x^2 + 21.559x + 1.5042$ | 0.95 |
| Free GSNO | | Fig 5 D. | 11.334 | 0.00* | 0.3737 | 0.8867 | $y = -2.5556x^2 + 21.361x + 2.6588$ | 0.97 |
| NP Al-GSH | | - | 1.768 | $0.17^{ns}$ | 0.2822 | 0.2332 | - | - |
| NP CS-GSNO | Root dry mass | Fig 5 E. | 3.902 | 0.01* | 0.3990 | 0.3315 | $y = -0.0033x^2 + 0.039x + 0.0139$ | 0.69 |
| | Shoot dry mass | - | 1.805 | $0.16^{ns}$ | 0.5640 | 0.2604 | - | - |

| | | | | | | | | |
|---|---|---|---|---|---|---|---|---|
| NP Al-GSNO | Root dry mass | - | 1.606 | 0.20 ns | 0.0607 | 0.0657 | - | - |
| | Shoot dry mass | Fig 5 F. | 3.342 | 0.02* | 0.8411 | 0.7392 | $y -0.0112x^3 + 0.1162x^2 - 0.3376x + 0.4532$ | 0.65 |
| NP CS-GSH | Root dry mass | - | 2.452 | 0.07ns | 0.4469 | 0.8016 | - | - |
| | Shoot dry mass | - | 2.036 | 0.12 ns | 0.7119 | 0.2212 | - | - |
| NP Al-GSH | Root dry mass | - | 0.932 | 0.44 ns | 0.3150 | 0.0570 | - | - |
| | Shoot dry mass | - | 2.611 | 0.06 ns | 0.9493 | 0.4485 | - | - |
| Free GSNO | Root dry mass | - | 2.353 | 0.08 ns | 0.0959 | 0.1347 | - | - |
| | Shoot dry mass | - | 1.990 | 0.12 ns | 0.2594 | 0.0927 | - | - |
| | | | | | | | | |
| NP CS-GSNO | | Fig 6. | 96.401 | 0.00* | 0.0603 | 0.9835 | $y = -0.1632x^2 + 4.5099x + 13.828$ | 0.99 |
| NP CS-GSH | | | 15.934 | 0.00* | 0.6763 | 0.2589 | $y = -0.1692x^2 + 4.6093x + 12.179$ | 0.95 |
| NP Al-GSH | | | 188.698 | 0.00* | 0.0510 | 0.3765 | $y = -0.177x^2 + 4.684x + 15.318$ | 0.96 |

| | | | | | | | | |
|---|---|---|---|---|---|---|---|---|
| NP Al-GSNO | | | 74.361 | 0.00* | 0.557 | 0.2449 | $y = -0.1657x^2 + 4.359x + 18.136$ | 0.99 |
| Free GSNO | | | 175.460 | 0.00* | 0.2551 | 0.9782 | $y = -0.172x^2 + 4.6588x + 15.024$ | 0.99 |
| Water | | | 265.494 | 0.00* | 0.4818 | 0.5296 | $y = -0.1439x^2 + 4.4097x + 15.249$ | 0.98 |
| | | | | | | | | |
| - | | Fig 7 A. | 13.024 | 0.00* | 0.3908 | 0.2153 | - | - |
| - | | Fig 7 B. | 28.315 | 0.00* | 0.7245 | 0.8696 | - | - |
| | | | | | | | | |
| - | | Fig 8 A. | 5.633 | 0.00* | 0.1326 | 0.9164 | - | - |
| - | | Fig 8 B. | 4.395 | 0.00* | 0.8581 | 0.7735 | - | - |
| - | | Fig 8 C. | 4.890 | 0.00* | 0.6669 | 0.1522 | - | - |
| - | | Fig 8 D. | 7.362 | 0.00* | 0.0993 | 0.1375 | - | - |
| - | | Fig 8 E. | 3.255 | 0.02* | 0.3227 | 0.5514 | - | - |
| - | | Fig 8 F. | 2.571 | 0.05* | 0.1414 | 0.5894 | - | - |
| | | | | | | | | |
| - | Shoot fresh mass | Fig S1 A. | 1.449 | $0.24^{ns}$ | 0.4501 | 0.5823 | - | |
| - | Root fresh mass | Fig S1 A. | 0.582 | $0.75^{ns}$ | 0.2522 | 0.2524 | - | |

| | | | | | | | | |
|---|---|---|---|---|---|---|---|---|
| - | Nodule fresh mass | Fig S1 A. | 1.895 | $0.13^{ns}$ | 0.4177 | 0.6895 | - | - |
| - | Total number of nodules | Fig S1 B. | 1.925 | $0.12^{ns}$ | 0.1448 | 0.3726 | - | - |
| - | Shoot length | Fig S1 C. | 1.408 | $0.25^{ns}$ | 0.6517 | 0.5044 | | |
| - | Root length | Fig S1 C. | 1.207 | $0.33^{ns}$ | 0.005 | 0.2962 | | |
| - | Shoot dry mass | Fig S1 D. | 1.802 | $0.15^{ns}$ | 0.7252 | 0.2238 | - | - |

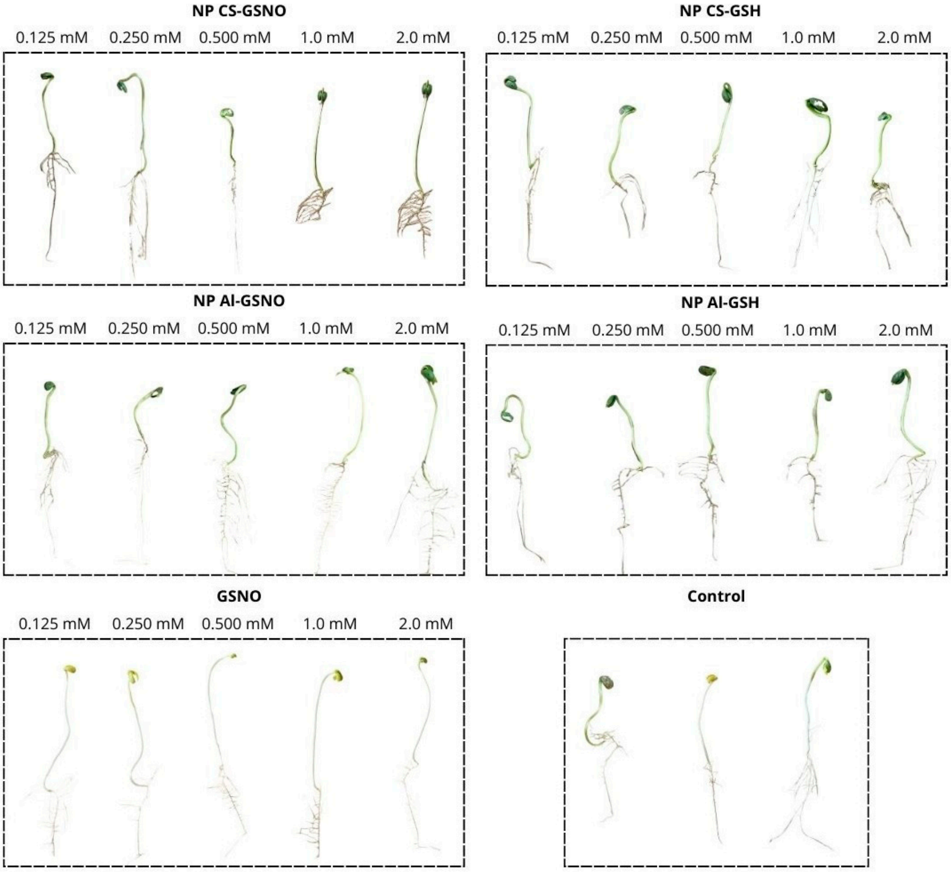


**Figure S1.** Representative images of *Glycine max* (L.) Merr. seedlings from the treatments: nanoparticles with chitosan nanoparticles with and without NO (NP CS-GSNO and NP CS-GSH), alginate nanoparticles with NO (NP Al-GSNO), free GSNO, and control (water). Concentrations of 0.125 mM, 0.250 mM, 0.500 mM, 1.0 mM, and 2.0 mM of GSNO/GSH were used.

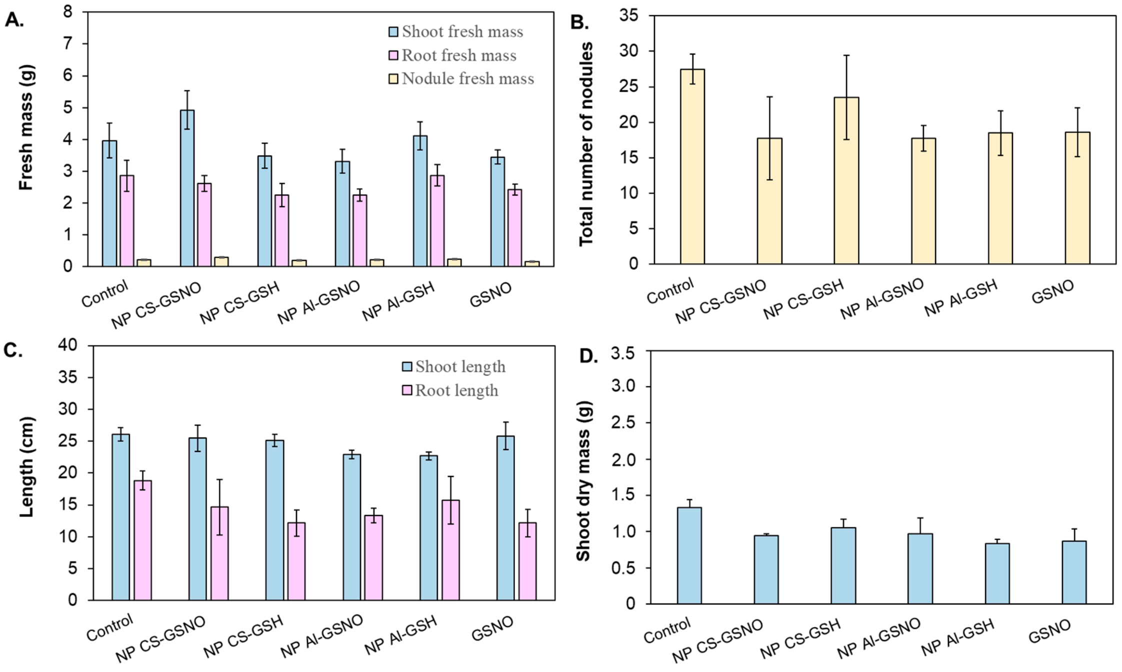


**Figure S2**. Effect of seed treatment with chitosan nanoparticles with and without NO (NP CS-GSNO and NP CS-GSH), alginate nanoparticles with and without NO (NP Al-GSNO and NP Al-GSH), and GSNO on plant growth. A concentration of 1.0 mM of GSNO/GSH was used in this experiment. The control treatment refers to seeds treated with distilled water only. The following variables were measured for *Glycine max* (L.) Merr. plants grown in a greenhouse: fresh mass (A), total nodule number (B), length (C), and shoot dry mass (D). The values presented represent the mean of six replicates ± the standard error. No significant difference was detected based on the results of the ANOVA followed by the Scott-Knott test ($p \leq 0.05$).

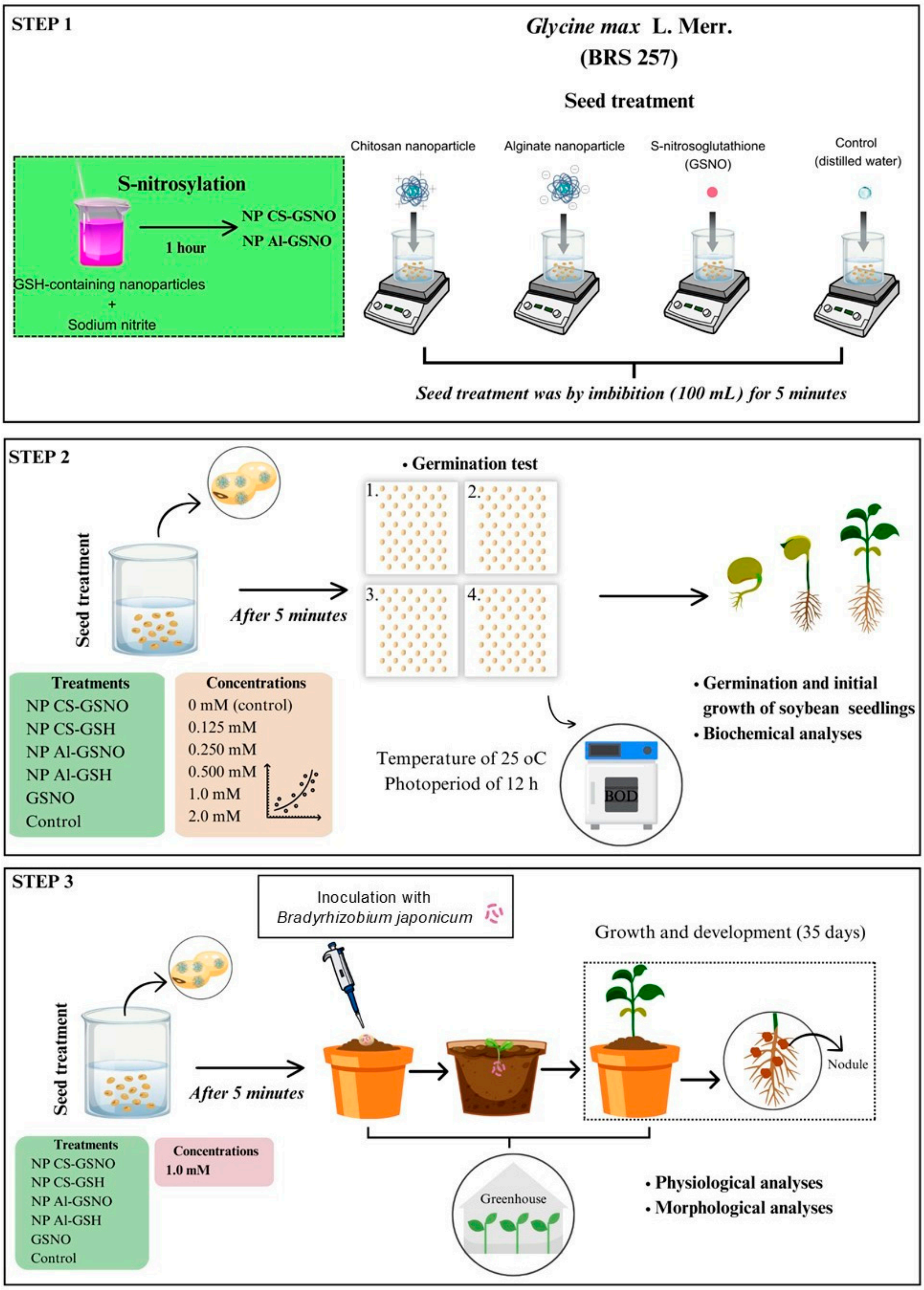


**Figure S3**. Representative scheme of the treatments of the seeds. *Step 1* refers to the nitrosation of nanoparticles with sodium nitrite ($NaNO_2$) and the seed treatment by immersion. *Step 2* covers the procedures conducted during germination and early seedling growth experiments. *Step 3* involves the procedures carried out during the nodulation experiment.
